# The Foreign Journals: Midwifery

**Published:** 1841-04

**Authors:** 


### MIDWIFERY.

*Cæsarean Operation saving the Lives of both Mother and Child.*
By Dr. Königsfeld, of Aachen.

At noon on the 28th March I was called to a woman in labour who, in consequence of a remarkable narrowness of the pelvis, had had already for several days severe but ineffectual pains. I found that she was small and bore various signs of having suffered from rickets. The pains were very severe; the woman could scarcely stand; the liquor amnii had not yet escaped. External examination discovered a remarkable bending inwards of the sacrum, and a furrow, nearly an inch and a half deep, formed by the projecting forwards of the two last lumbar vertebræ. The ossa pubis diverging downwards were so much pressed in upon the abdomen above, that all the walls of the abdomen above them hung over them like a sack. The vagina was hot and very narrow; the os uteri as large as a dollar, and very hard; the membranes were moderately tense, and the child's head could be obscurely felt behind them, in the same position as, according to the midwife, it had occupied for the last four days. An accurate measurement showed that the conjugate diameter of the pelvis was only between two and two inches and a half long, and I therefore determined on the Cæsarean operation as the only means of effecting delivery; for, even if perforation and embryotomy could have been performed, they were forbidden by the child being still alive, and the patient rapidly sinking.

The incision was commenced half an inch on one side of the umbilicus, was then turned to the linea alba, and, extending for six inches, terminated close to the os pubis. The uterus presented at the wound, and was divided in the same direction as the external incision, and opened for an inch at its upper part. The membranes were now broken somewhat lower down, carrying the finger along on the knife, and the waters were allowed to flow *per vaginam;* the wound in the uterus was then carried on, and the membranes separated lower down. The right shoulder of the child now pressed into the wound, and was slowly pushed on, bringing after it the right arm, the chest, the buttocks, and the lower extremities; the head stuck in the wound, and required great care in removing it. The intestines, which were forcibly pushed forwards, were held back by large sponges, and the placenta was removed with the hand as soon as possible. The hemorrhage, throughout the operation, was inconsiderable; and after as much as possible of that which had flowed, had been pressed out of the abdomen, the wound was united by suture and plaster. The operation lasted twelve minutes. Four hours after, bloodletting was found necessary to calm the violent inflammatory excitement that had commenced; but after this, with only mild treatment and perfect abstinence (for hunger, says the author, is the best antiphlogistic,) the case went on perfectly well; the wound was healed, and the patient entirely recovered four weeks after the operation. The child also was healthy from its birth, and grew very rapidly.

*Casper's Wochenschrift. December* 5, 1840.

---

*Singular Case of Tumour in the Pelvis.* By Professor von D'OUTREPONT, of Würzburg.

A WOMAN, 26 years old and well made, gave birth when 25 years of age to her first child without difficulty. Towards the end of her second pregnancy she again applied at the hospital in consequence of experiencing pain in the pelvic region. Vaginal examination discovered a hard and painful tumour, extending from the inner surface of the left ischium nearly to the corresponding point on the opposite side. It was hard, globular, even on its surface, and occupied the ascending ramus of the ischium and the descending ramus of the pubis, and extended over the obturator foramen. It was impossible to reach the lower segment of the uterus, or to feel any part of the child.

The size and hardness of the tumour seemed to leave no chance of the birth of a living child, even by the induction of premature labour. Professor D'Outrepont, who doubted whether the tumour was fibro-cartilaginous or a true bony exostosis, asked the opinion of many eminent men who saw the case. They did not express themselves with certainty as to its nature, and the patient refused to allow an experimental incision to be made into the tumour.

A short time before labour began, the tumour was thought to have become slightly compressible. When labour commenced, the professor called a consultation in which it was determined that unless a great change had taken place in the character of the tumour, an attempt should be made to remove it, or to cut away the bone if that should be found to be implicated, and as a last resource to perform the Cæsarean section.

On an examination being made, the right foot of the child was found to present, the cord was prolapsed, and did not pulsate. The tumour, however, was found to be so much softened that it was possible to pass three fingers through the outlet of the pelvis. Professor D'Outrepont brought down the foot, in doing which he found that the hips had compressed the tumour still more. The chief difficulty was experienced in extracting the head by means of the forceps, which gave the patient considerable pain. The child was still-born but was speedily recovered. After the birth of the child the tumour regained its former size, so that the placenta could not be expelled by the natural efforts, and it was necessary to introduce the hand in order to remove it.

The patient recovered rapidly, and returned ten weeks after her delivery, in order to have the tumour removed, which operation was performed by Professor Textor. The growth was found to be fibro-cartilaginous, and was connected neither with the bone nor the periosteum. It weighed 11½ ounces, and was so hard that none but they who were present at the patient's delivery, could have believed its previous softening possible. The patient was completely cured.

*Neue Zeitschrift für Geburtskunde. Band* ix, *S.* 1.

